# Extracellular matrix stiffness activates mechanosensitive signals but limits breast cancer cell spheroid proliferation and invasion

**DOI:** 10.3389/fcell.2023.1292775

**Published:** 2023-12-06

**Authors:** Irina Jahin, Thomas Phillips, Stefania Marcotti, Mark-Alexander Gorey, Susan Cox, Maddy Parsons

**Affiliations:** Randall Centre for Cell and Molecular Biophysics, King’s College London, London, United Kingdom

**Keywords:** breast cancer, mechanotransduction, invasion, extracellular matrix, YAP, ERK

## Abstract

Breast cancer is characterized by physical changes that occur in the tumor microenvironment throughout growth and metastasis of tumors. Extracellular matrix stiffness increases as tumors develop and spread, with stiffer environments thought to correlate with poorer disease prognosis. Changes in extracellular stiffness and other physical characteristics are sensed by integrins which integrate these extracellular cues to intracellular signaling, resulting in modulation of proliferation and invasion. However, the co-ordination of mechano-sensitive signaling with functional changes to groups of tumor cells within 3-dimensional environments remains poorly understood. Here we provide evidence that increasing the stiffness of collagen scaffolds results in increased activation of ERK1/2 and YAP in human breast cancer cell spheroids. We also show that ERK1/2 acts upstream of YAP activation in this context. We further demonstrate that YAP, matrix metalloproteinases and actomyosin contractility are required for collagen remodeling, proliferation and invasion in lower stiffness scaffolds. However, the increased activation of these proteins in higher stiffness 3-dimensional collagen gels is correlated with reduced proliferation and reduced invasion of cancer cell spheroids. Our data collectively provide evidence that higher stiffness 3-dimensional environments induce mechano-signaling but contrary to evidence from 2-dimensional studies, this is not sufficient to promote pro-tumorigenic effects in breast cancer cell spheroids.

## 1 Introduction

Solid tumors exist within complex three-dimensional (3D) extracellular matrices (ECM) comprised of a complex network of collagens with other constituent macromolecules such as fibronectin, hyaluronic acid and proteoglycans (Mohan et al., 2020). Cells sense biophysical properties of the surrounding ECM through integrins and other surface receptors driving downstream signaling pathways and transcription (Cooper and Giancotti, 2019). These mechano-sensitive signaling pathways control a range of functional outcomes including mitotic signaling and cytoskeletal re-organization to promote invasion through the surrounding matrix (Yamada and Sixt, 2019). Increased ECM stiffness can occur during tumor progression (Handorf et al., 2015), with *in vitro* data supporting roles for these changes in control of cancer cell motility and proliferation (Wullkopf et al., 2018). The pathogenic role of increasing stiffness within the tumor microenvironment has been particularly well documented in breast cancer progression, with higher ECM stiffness correlating with poor prognosis and drug resistance (Provenzano et al., 2008; Levental et al., 2009; Boyd et al., 2014; Acerbi et al., 2015; Lindstrom et al., 2015; Joyce et al., 2018).

Several key mechanosensitive signaling pathways have been identified as potential regulators of cancer cell responses to increasing ECM stiffness. The transcription factor YAP is perhaps the best studied of these pathways, with its activation triggered by extracellular stiffness leading to downstream transcriptional regulation. YAP (and its paralog TAZ) are central, overlapping, but distinct components of the Hippo signaling pathway that controls cell fate, cell growth, organ size, and morphogenesis (Davis and Tapon, 2019). The core pathway functions with MST1/2 and binding partner SAV1 activating LATS1/2 kinases and their scaffold MOB1A/B. Activated LATS kinase then phosphorylates and inactivates YAP/TAZ. Phosphorylated YAP/TAZ is sequestered in the cytoplasm through interaction with 14-3-3 proteins or is subsequently ubiquitinated and degraded by the proteasome (Cai et al., 2021). YAP/TAZ activity promotes interaction with TEAD transcription factors in the nucleus to induce expression of a plethora of downstream targets which promote proliferation, cell survival and ECM remodeling (Davis and Tapon, 2019). Biomechanical regulation of YAP activity by cell-cell junctions, focal adhesions and by the actin cytoskeleton have all been suggested to play roles in tumourigenesis (Moreno-Vicente et al., 2019), but contributions of YAP activity to breast cancer progression remain controversial (Lee et al., 2019; Fresques and LaBarge, 2020; Qin et al., 2020).

Emerging evidence also suggests that the MAP kinase (MAPK) signaling pathways are important mechano-sensitive proteins that may play a role in stiffness sensing in cancer. Oncogenic mutations to MAPK signaling components have been well studied as contributing to uncontrolled growth and tumorigenesis (Braicu et al., 2019). ERK1/2 is one family member that typically integrates growth factors and mitogens to induce cell growth and differentiation (McKay and Morrison, 2007). The ERK signaling cascade is triggered by the small GTPase Ras, which is activated in response to mitogen stimulations of receptor tyrosine kinases at the plasma membrane. Once phosphorylated, ERK enters the nucleus and alters gene expression by phosphorylating transcription factors such as Elk1, Ets-2 and c-Myc in addition to cytoplasmic substrates (Unal et al., 2017). ERK has been suggested to be mechano-responsive downstream of stiffness-induced receptor tyrosine kinase activation (Hwang et al., 2015; Zhang et al., 2017; Yang et al., 2018; Farahani et al., 2021). Moreover, MEK inhibitors have been suggested to specifically block stiffness induced drug resistance in breast cancer cells (Schwartz et al., 2017). Interestingly, several lines of evidence suggest that YAP and ERK may be co-regulated by extracellular stiffness in cancer. A reduction in the YAP target Cyr61 leads to reduced ERK phosphorylation and breast cancer cell invasion (Hellinger et al., 2019). ERK has also been shown to be upstream of YAP in breast cancer cell lines subjected to low shear stress (Qin et al., 2019). Conversely, ERK phosphorylation is reduced following YAP knockdown in colon cancer cell lines (Liu et al., 2019) suggesting a complex regulatory axis may be in play. Evidence for ERK/YAP synergy can also be found in ovarian cancer (Shi et al., 2020) and melanoma (Yu et al., 2015) cell lines.

Whilst these and other studies all point towards extracellular stiffness in regulating cancer cell signaling, much of the analysis to date has been performed in cells plated on 2D surfaces where stiffness is varied using polymer-based approaches. Whilst these are useful tools to define mechano-signaling, the 2D stiffnesses ranges used are often well above the physiological levels seen in disease settings, and do not recapitulate the complexity of the 3D topographical or structural features seen in the tumor microenvironment (Micalet et al., 2023). Moreover, these studies are largely restricted to assessing single cells in contact with the substrate; however, this is not the case in solid tumors, where most cells only exhibit cell-cell interactions. The emerging differences in mechano-sensing in cells on 2D surfaces vs. in 3D and the known impact of this on signaling and functional outcomes remain important issues in the field, particularly in the context of drug discovery (Van Zundert et al., 2020).

Here we present evidence that both ERK and YAP are activated in MCF7 and HCC1954 human breast cancer cell spheroids in 3D collagen scaffolds of increasing stiffness. Both transcriptional regulators showed higher activity in outer spheroid cells in contact with the ECM compared to those within the spheroid, only exhibiting cell-cell contact. Blocking both EGFR and collagen-binding integrins resulted in reduced ERK activity in both inner and outer cells within spheroids. Inhibition of ERK resulted in reduced YAP activity and reduced YAP-dependent invasion, indicating these two mechanosensitive pathways are intricately connected. Activity of both ERK and YAP were required for proliferation and invasion in low stiffness 3D scaffolds, and YAP was sufficient to induce invasion in MCF7 cells in this setting. However, higher stiffness ECM reduce F-actin based protrusion, invasion and proliferation in both cell lines, anti-correlating with ERK and YAP activation. Our findings demonstrate that stiffening of 3D matrices suppresses proliferation, protrusion, and invasion of breast cancer cells despite activation of mechanically sensitive pathways that might be expected to promote these phenotypes. Our data highlight the importance of analyzing mechano-sensitive pathways in contact and pave the way for studying longer-term impacts of stiffness-induced transcriptional changes that may mediate tumor progression and drug response in cancer.

## 2 Methods

### 2.1 Antibodies

Primary antibodies used were ERK1/2 and ppERK1/2 (Cell Signaling Technology; #9102 and #9101 respectively), GAPDH (GeneTex; GTX627408), anti-species Horseradish Peroxidase (HRP)—conjugated secondary antibodies (Dako), AlexaFluor-conjugated secondary antibodies (Thermo Fisher Scientific), Hoechst 33342 (Sigma-Aldrich; #T5840), YAP (Santa Cruz Biotechnology; #sc-271134), Ki67 (Abcam; # ab15580), DAPI (Sigma-Aldrich; #D9542), E-cadherin (Thermo Fisher Scientific; # 13-1700), Alexa-dye labelled phalloidin (Thermo Fisher Scientific).

### 2.2 cDNA constructs and siRNA

GFP**-**LifeAct and mScarlet-LifeAct lentiviral constructs were as previously described (Scales et al., 2013; Phillips et al., 2023), pEGFP-N1 was purchased from Clontech, pEGFP-C3-hYAP1 was a gift from Marius Sudol [Addgene plasmid # 17843; http://n2t.net/addgene:17843; RRID:Addgene_17843 (Basu et al., 2003)], pENTR-ERKKTRClover was a gift from Markus Covert [Addgene plasmid # 59138; http://n2t.net/addgene:59138; RRID:Addgene_59138 (Regot et al., 2014)] and was subcloned into the lentiviral backbone pLX302 using the Gateway cloning system (Invitrogen, Paisley, United Kingdom), YAP ON-TARGETplus SMARTpool siRNA was purchased from Horizon Discovery (Cambridge, United Kingdom).

### 2.3 Cell culture

Two human breast carcinoma cell lines were used in this study: MCF7 and HCC1954. HCC1954 cells are highly invasive, express high levels of HER-2 but not estrogen receptor/progesterone receptor (ER/PR) (Gazdar et al., 1998). Conversely, MCF7 cells are non-invasive, express low levels of HER-2 and express both ER and PR (Bacus et al., 1990; Ziegler et al., 2014). Both cell lines exhibit epithelial-like morphology in culture. MCF7 cells were cultured in EMEM (Sigma-Aldrich), HCC1954 were cultured in RPMI-1640 (Sigma-Aldrich) and HEK293T were grown in high glucose DMEM (Sigma-Aldrich). All cells were originally purchased from ATCC. All media were supplemented with 10% v/v heat-inactivated FBS, 2 mM L-Glutamine, 100 units/mL penicillin and 10 mg/mL streptomycin (all from Sigma-Aldrich). Cells were maintained at 37°C in a humidified environment and 5% CO_2_.

### 2.4 cDNA and siRNA transfection

Cells were plated at 30%–40% confluence 24 h prior to transfection, which was performed using Lipofectamine-3000 (Thermo Fisher Scientific) transfection reagent, according to manufacturer instructions. DNA-Lipofectamine mix was added dropwise to cells, transfection medium was replaced with complete culture medium after 6 h. Where appropriate, stable cell line transfection was achieved using 0.88 mg/mL G418 selection reagent (Roche) in complete cell culture medium to select for construct expression. These stable cells were maintained in 0.88 μg/mL G418-inclusive full culture medium to maintain target expression. For siRNA-mediated knockdown, cells were transfected using DharmaFECT1 (Horizon Discovery) reagent according to manufacturer instructions. Following incubation, siRNA mix was added dropwise to cells followed by 6–8 h incubation. siRNA-opti-MEM mixture was aspirated and replaced with complete culture media. siRNA-mediated knockdown was verified by Western blotting control vs knockdown conditions and consistent target knockdown was demonstrated for the entirety of experiment timespans.

### 2.5 Lentiviral transduction

HEK293-T cells were plated in a T25 flask at 50%–60% confluence 24 h prior to transfection with lentiviral constructs. In 500 µL opti-MEM, 3.75 µg lentiviral construct, 2.8125 µg p∆8.91 and 0.9375 µg pMDG were combined and vortexed before addition of 22.5 µg PEI transfection reagent. This mixture was vortexed and left to incubate at RT for 15 min. Meanwhile, HEK293-T medium was replaced with 1.5 mL opti-MEM and, post-incubation, the 500 µL transfection mixture. Medium was changed after 4 h to opti-MEM and harvested after 48 h. This virus-rich medium was filtered with 0.45 µm syringe filter to remove cell debris. 1 mL viral medium was added to cell lines plated at 50%–60% confluence in T25 flask with 8 μg/mL Polybrene (Sigma-Aldrich) in full culture medium. Medium was replaced with full culture medium after 48 h followed by assessment of transduction efficiency.

### 2.6 Western blotting

Western blotting was used for specific protein detection following SDS-PAGE. The proteins were transferred onto PVDF membranes (Sigma-Aldrich) and blocked using 5% BSA/0.1% Tween20 (Sigma-Aldrich) in Tris Buffered Saline (TBS; TBST) or 5% milk/TBST for 1 h at room temperature. Membranes were then probed with the primary antibody at 1:1000 dilution overnight at 4°C and washed prior to incubation with HRP-conjugated secondary antibodies for 1 h at room temperature. Proteins were detected with ECL chemiluminescence kit (Bio-Rad Laboratories) and imaged (ChemiDoc Imaging Systems, Bio-Rad Laboratories). Blots were analyzed and processed using Image Lab (v5.2.1, Bio-Rad Laboratories).

### 2.7 Spheroid formation and embedding

Spheroids were formed and embedded as in (34). Briefly, MCF7/HCC1954 spheroids of 400 and 750 cells, respectively, were generated using the hanging drop method with a 1:3 ratio of methylcellulose:cell culture medium with spheroids forming between 24 and 72 h. Collagen matrices were composed of 2 mg/mL rat tail collagen I (Corning; # 354249), Opti-MEM (Gibco), 1 mg/mL Fibronectin (Merck Millipore), 20 mM HEPES (Sigma-Aldrich), 0.3% w/v sodium bicarbonate (Sigma-Aldrich) and 4 mM sodium hydroxide (Sigma-Aldrich). Where ribose was used to stiffen collagen matrices, ribose was incubated with an appropriate volume of collagen for 30 min on ice before adding other components and plating gels. Ribose was added to give final concentrations of 50 or 200 mM to provide increased matrix stiffness. Previous measurements using atomic force microscopy (AFM) defined the stiffness of 0 mM ribose gels as 0.2 kPa, 50 mM as 0.4 kPa and 200 mM as ∼1.2 kPa, which lie in the physiological range for human breast cancer (Plodinec et al., 2012). Stiffness variation using ribose has been shown to have no effect on matrix viscoelasticity (Roy et al., 2010) and increase collagen bundle width without affecting pore size (Mason et al., 2013). Where Cy3-stained collagen was used, a monoreactive Cy3 staining kit (Cytiva) was used to stain 1 mg of stock collagen for use at a 1:3 ratio stained:unstained collagen, with this mixture pipetted slowly up and down to achieve homogeneity. Following collagen staining, stained collagen stock was dialyzed using an 8 kDa molecular weight cutoff dialysis kit (Cytiva) twice in 0.1% acetic acid in ddH_2_O for 2 h and overnight, and once in 0.1% acetic acid in PBS at 4°C. All collagen matrix formulation was carried out in tubes kept on ice. Collagen matrices of 2 mg/mL final collagen concentration were stamped with PDMS stamps featuring 200 µm diameter and 250 µm height micro-pillars to create 200 µm microwells for spheroid embedding. Stamps were placed on top of Mylar film supports (Radioshack Pro) of varying thickness to determine coverslip-spheroid Z-distance with this distance determined by objective lens working distance. Stamped collagen matrices were placed in a cell culture incubator at 37°C for 2 h to polymerize, after which stamps were removed and cell culture medium was added on top of the matrix. Spheroids were added manually by pipetting from hanging drop under a dissecting microscope in a class II, type A1 biosafety hood. For most experiments, 2- or 4-chamber µslides (Ibidi) were used for collagen stamping and spheroid imaging. A minimum of 4 spheroids were added to individual chambers for each condition. For inhibitor treatments, U0126 (10 μM, 2 h; ERK inhibitor), AG1478 (5 μM, 4 h; EGFR tyrosine kinase inhibitor) or BTT3033 (10 μM, 4 h; α2β1 integrin inhibitor) (all purchased from Tocris; #1144, #1276 and #4724 respectively) were incorporated into the media prior to imaging. Inhibitor treatments were refreshed at 48 h.

### 2.8 Atomic force microscopy (AFM) collagen-ribose imaging and analysis

Cy3-labeleld collagen gels with or without ribose were cast into Ibidi 35 mm μ-Dishes with a polymer coverslip and allowed to polymerise before washing with PBD and replacing with OptiMEM. Samples were imaged live to avoid artefacts of additional crosslinking and changes in sample stiffness. AFM images were obtained using Nano Wizard 3 AFM (JPK Instruments). The whole AFM system was kept in an isolation cupboard, a Halcyonics i4 vibration isolation system, to minimise the effects of ambient vibrations on the readings obtained from the cantilever tip. The cantilevers used in all AFM experiments were SD-qp-SCONT-TL-10 with a PPMA bead at 5 μm radius glued to the end with an approximate spring constant of 0.013 N/m taken in liquid prior measurements. This enables the measurement of the collagen meshwork as opposed to individual fibres. Images were recorded on quantitative imaging (QI) mode, which records a complete force-distance curve for each pixel without exerting lateral forces on the sample. The set point was set at 1 nN, 10 μm Z length at a 2 μm/s extension speed. The cantilevers were calibrated by measuring the thermal fluctuations within. Young’s Modulus of collagen-ribose matrices were extracted by Hertz model fitting method on the top 10% of the AFM indentation curve using the JPK AFM software. Hertz fitting was performed on the top 10% of the extension curve to remove indentations along the curve when breaking past different viscoelastic barriers of the matrix from model fitting calculations. Each curve was visually checked to verify a clean contact point, extension, and retraction curve with no interference from unpolymerized collagen debris or fiber attachments.

### 2.9 Immunofluorescence and microscopy

For fixed samples, a standard protocol was followed for immunofluorescent staining and samples were covered with foil to limit light exposure. Samples were washed once with PBS for 15 min then fixed for 2 h with 10% formalin solution (Sigma-Aldrich). Samples were then washed for 3 × 10 min with PBS and permeabilized with 0.25% Triton-X100 (Sigma-Aldrich) in PBS (PBST) for 15–30 min on a rocker. Samples were then washed thrice for 10 min each with PBS followed by blocking for minimum 1 h on a rocker in 5% bovine serum albumin (BSA, Roche) in PBST. Following blocking, primary antibody/dye was added in 5% BSA PBST overnight on a rocker at 4°C. If secondary staining was required, samples were washed 3 × 10 min in PBS and secondary antibody was added for a minimum incubation of 1 h. Samples were kept covered at 4°C until imaging.

Imaging was carried out using Nikon A1R point-scanning confocal microscopy (PSCM), Yokogawa SoRa spinning disk microscopy or Zeiss LSM 880 multiphoton microscopy. For PSCM and SoRa microscopy, standard laser lines of 405, 488, 561, and 647 nm were used, while multiphoton microscopy used 488 and 561 nm laser lines for imaging fluorophores with ablation carried out in manually designated ROIs using a pulsed Chameleon Vision II TiSa laser (Coherent) tuned to 760 nm at 100% for three repeats at 15 ms per repeat. Invasion assays imaged the whole sample thickness with 10 µm Z-slices for each spheroid (GFP-expressing) embedded in Cy3-stained collagen once per day for 5 days using a ×20 air objective. Depending on spheroid invasiveness, spheroids were embedded at either 125 µm (MCF7 spheroids) or 250 µm (HCC1954 spheroids) above the coverslip. Filopodia were imaged on the SoRa using 0.3 µm Z-slices within a 10 µm total Z-stack in mScarlet-LifeAct-expressing MCF7 GFP-YAP spheroids 24 h post-embedding 75 µm above the coverslip using a ×40 water objective. ECM timelapse movies of HCC1954 LifeAct-GFP spheroids embedded 125 µm above the coverslip in Cy3-stained collagen matrices were captured on the SoRa 24 h post-embedding at an axial resolution of 0.5 µm in a total Z-stack of 25 µm using a ×40 water objective. Timelapse imaging captured at an interval of 5 min for 30 min total.

For ablation experiments, Samples were imaged live on a Zeiss LSM 880 microscope with Airy scan using an LD C-Apochromat 40x WI Korr M27 objective (NA 1.1) using 1x zoom with excitation wavelengths of 488 nm (argon laser) and 561 nm (DPSS 561-10 laser) used. For ECM ablation, regions of interest were manually defined at periphery of the spheroid at least 20 μm from the spheroid. A pulsed Chameleon Vision II TiSa laser (Coherent) tuned to 760 nm was used at 100% for three repeats at 15 ms per repeat. Spheroids were imaged to generate a 144 μm region in z composed of 72 z-slices per spheroid. Images were captured pre-ablation and post-ablation, with time-lapse imaging performed post-ablation with a temporal resolution of 10 min for 30 min total. Imaging parameters were optimised to reduce phototoxicity by setting laser power with lookup tables set to 50% of maximum dynamic range. Images were output as. czi format and image acquisition performed using Zeiss ZEN (version 1.0) with post-processing occurring in Fiji.

For proliferation assays, spheroids were embedded in separate gels and fixed at 24 and 96 h before staining with DAPI and imaging the whole sample thickness with Z-slices every 4 µm using a ×40 water objective. For YAP nuclear:cytoplasmic ratio quantification, samples were fixed and stained 24/72/96 h post-embedding with DAPI, phalloidin and, in HCC1954 spheroids, anti-YAP with AlexaFluor-conjugated secondaries. Spheroids were imaged from 5 µm below the spheroid to the top of the spheroid with 1 µm Z-slice using a ×40 water objective.

### 2.10 Invasion and protrusion analysis

Invasion analyses compared differences in spheroid channel area and the number of broad multicellular protrusions (defined as more than three collectively invading cells) over 5-day invasion time-courses. In Fiji, Z-stacks of the spheroid channel were maximum intensity Z-projected, then thresholded to generate a single central area which represented the total extent of spheroid invasion into the collagen-ribose gel. The Particle Analyzer tool was then set to a size bracket between 20000 and infinity μm^2^ to exclude background pixels and cell debris and binarized to calculate total thresholded region area. Spheroid area was calculated relative to the initial timepoint (hour 0) to provide a relative measurement of spheroid area change over time. Broad multicellular protrusions were manually counted for each spheroid using both spheroid and nuclear channels.

For filopodia analysis, mScarlet-LifeAct channels were extracted and 12 μm maximum intensity projections generated for analysis. Regions of interest were analysed using the FiloQuant plug-in (43). Briefly, regions of interest at the spheroid boundary were used to generate thresholded regions of the spheroid body. Skeletonized structures were then generated and used in conjunction with the body threshold to identify filopodia-like structures. From these analyses, individual filopodia length was measured (with a minimum size threshold used to remove background) and used to calculate mean filopodia length.

### 2.11 Proliferation analysis

Bulk spheroid proliferation was analyzed using the DAPI channel and creating maximum intensity Z-projections equivalent to 5 µm sections in two distinct regions of the spheroid with a minimum of 18 images analyzed per condition. Briefly, the DAPI channel was extracted to show nuclei and two different non-overlapping Z-slice regions were used to generate maximum intensity projections representing a 5 μm region of the spheroid. These images were then analyzed using QuPath (Bankhead et al., 2017) where images were annotated using the magic wand tool followed by cell detection to identify individual nuclei. Resulting detections were analyzed using the 2D Delaunay Clustering analysis tool that measures distances between the center of each nuclei, to determine mean neighbor distance and mean number of neighbors.

### 2.12 ERK/YAP image analysis

ERK and YAP nuclear:cytoplasmic ratio was calculated by drawing one 10 × 10 pixel square in the cell nucleus (designated by overlap with DAPI signal) and two 10 × 10 pixel squares in the cytoplasm (with cell-cell boundary shown with phalloidin staining). The mean pixel intensity was taken from these regions, and nuclear signal was divided by cytoplasmic signal, relative to background measurements, to provide a cell by cell nuclear:cytoplasmic ratio reading. ERK-KTR cytoplasmic:nuclear ratio was similarly measured, however, the cytoplasmic signal was divided by the nuclear signal. Where spheroids were stained with antibodies to Ki-67, each nucleus was scored as Ki-67 positive or negative, and recorded relative to ERK-KTR C:N ratio in the same cell. Per spheroid, a minimum of 24 measurements were taken from each of the outer and inner compartment with measurements taken from at least two distinct 5 µm maximum intensity projections in the Z-plane. Measurements were taken from a minimum of 9 spheroids total per condition from three independent repeats. YAP activity ablation assay measurements used a similar method, but individual cells were tracked over timelapse imaging and nuclear:cytoplasmic ratio was calculated before ablation and 30 min post-ablation in the same cells. Again, per spheroid, a minimum of 24 measurements were taken from each of the outer and inner compartment with measurements taken from at least two distinct 5 µm maximum intensity projections in the Z-plane.

### 2.13 E-cadherin linescan analysis

E-cadherin line scan analysis used the line tool in FIJI to draw a 10 µm long line perpendicular to maximum intensity Z-projected images of E-cadherin staining in spheroids with intensity histograms generated along this line. Values were normalized to distance 0 µm. A minimum of 12 junctions were quantified per image with at least 18 images analyzed per condition.

### 2.14 PIV and AFT analysis

Particle imaging velocimetry (PIV) analyses were carried out as in (45). Briefly, 350 × 700-pixel regions from timelapses composing part of the spheroid and surrounding ECM were used. A 2D cross-correlation was run by comparing a 5 µm-squared source windows within a 10 µm-squared search windows in the following time frame, with a minimum threshold of 0.3 on correlation to define retained source-search matches. A spatial convolution with a Gaussian kernel of 50 µm and sigma of 10 µm was applied to interpolate displacements. This analysis was based in MATLAB (MathWorks) and code is publicly available at https://github.com/stemarcotti/PIV.

Alignment by Fourier Transform (AFT) analyses were carried out as in (46). Briefly, 5 µm maximum intensity projections of Cy3-stained collagen and HCC1954 LifeAct-GFP spheroids were generated from the mid-spheroid region. AFT was used to generate orientation vectors over 25 × 25 pixel windows (with a 50% overlap) across the image, generating a vector field representing the local orientation of fibers. These local orientations were compared within neighbors of 5 × 5 vectors to generate an order parameter that correlates the directionality of vectors within the defined neighborhood, where values close to 1 representing high degree of neighborhood alignment and values near to 0 representing random alignment. Specifically, the order parameter is calculated as:

$$S=2\left(<\cos^{2}\theta_{ij}>-\frac{1}{2}\right).$$

with θ_ij_ representing the angle between the orientation of a central reference vector and its neighbors. AFT analyses were performed radially to the spheroid to quantify radial alignment in each cardinal and intercardinal direction.

### 2.15 Statistical analysis

Data are represented as mean ±25/75th percentile with min/max shown. All statistical tests were carried out using Prism package v9 (GraphPad). Data sets were tested for normality distribution using the Shapiro-Wilk test (for *n* < 50) or Kolmogorov–Smirnov test (for *n* > 50). Student’s t-test was performed for comparing two groups for statistical analysis. Analysis of variance (ANOVA) with Sidak *post hoc* test was used for multiple comparisons. *p* < 0.05 was considered as statistically significant.

## 3 Results

### 3.1 ERK activity increases with increasing ECM stiffness but anti-correlates with stiffness-dependent proliferation

**FIGURE 1:**
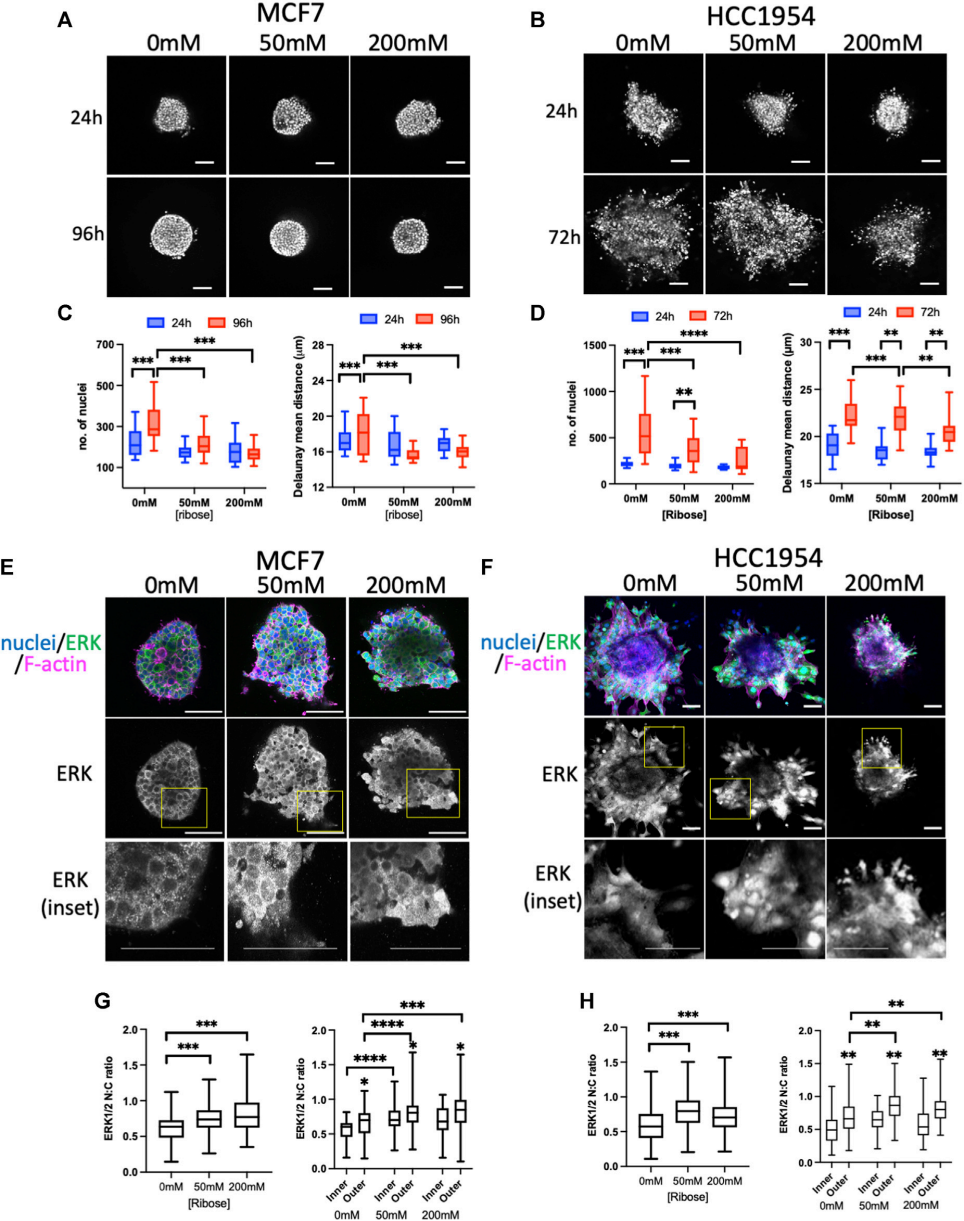
ERK activity anti-correlates with stiffness-dependent proliferation. **(A, B)** Representative images of MCF7 **(A)** and HCC1954 **(B)** cell spheroids embedded in collagen gels stiffened using ribose (0 mM, 50 mM and 200 mM). Live cells stained with Hoechst imaged every day over 96 h (MCF7) or 72 h (HCC1954). Images from 24 h and final time point shown. **(C, D)** Quantification of nuclei/spheroid (left graphs) and distance between nuclei (right graphs) in MCF7 **(C)** and HCC1954 cells. Data from 24 h (blue) and final time point (red) are shown **(D)**. **(E, F)** Representative images of MCF7 **(E)** and HCC1954 **(F)** cell spheroids embedded in collagen gels stiffened using ribose (0, 50, and 200 mM), fixed and stained for nuclei (blue), ERK (green) and F-actin (magenta). Boxed areas in ERK single channel panels shown as higher magnification inset panels below. **(G, H)** Quantification of nuclear:cytoplasmic (N:C) ratio of ERK with overall (left graphs) and inner vs outer cells (right graphs) for MCF7 **(G)** and HCC1954 **(H)**. Data shown is from one experiment with 10 spheroids per condition, representative of three independent experiments. Mean and 25/75th percentile with min/max are shown in graphs. **p < 0.01, ***p < 0.001, ****p < 0.0001. * without bars in G and H indicate differences between inner and outer cells. All other comparisons were not significant. Scale bars are 20 μm.

To assess stiffness-induced changes to signaling, 2 different human breast carcinoma cell lines were used in this study: MCF7 (non-invasive) and HCC1954 (invasive). Both cell lines readily formed spheroids, in-line with their epithelial-like growth characteristics. To explore potential mechano-sensitive proliferation responses, spheroids were embedded in collagen I gels within imaging chambers (Phillips et al., 2023) stiffened using the non-enzymatic crosslinker ribose. Similar to previous reports using non-enzymatic stiffening (Mason et al., 2013; Mason and Reinhart-King, 2013) images of sparsely labelled cy3-collagen fibers within these gels indicated no change in organization of fibers and this was confirmed by quantification of images using Alignment by Fourier Transform [AFT (Marcotti et al., 2021)] (Supplementary Figures S1A, B). Atomic force microscopy (AFM) analysis further confirmed increased stiffness with increasing ribose concentration within ranges previously described up to ∼1.2 kPa (Mason et al., 2013) (Supplementary Figure S1C). Daily imaging of live spheroids within these gels labeled with nuclear dyes (Figures 1A, B) revealed a significant increase in nuclear counts over time in both MCF7 cells (Figure 1C) and HCC1954 cells (Figure 1D) in unstiffened (0 mM) collagen gels compared to 50 and 200 mM conditions. Further analysis demonstrated that nuclei at the final time point of imaging were more closely packed together in higher stiffness scaffolds compared to lower in both cell lines (Figures 1B,D). Imaging of fixed spheroids stained for the cell-cell adhesion marker E-cadherin demonstrated that junctions between cells were not different between stiffnesses (Supplementary Figure S2A).

ERK is one of the critical known regulators of proliferation, and activity of this kinase has been suggested to be mechano-sensitive in cells on 2D surfaces (Hwang et al., 2015; Zhang et al., 2017; Yang et al., 2018; Farahani et al., 2021). We therefore aimed to understand whether ERK activity exhibited mechano-responsiveness in 3D and correlated with proliferation. Phosphorylation of ERK at Thr202/Tyr204 (ppERK) is a well-recognized indicator of activity of this kinase. Indeed, antibodies to ppERK readily detected changes in activity by Western blot upon serum starvation, EGF treatment or inhibition of ERK using U0126 (Supplementary Figure S2B). However, attempts to assess ppERK using immunostaining with a variety of commercial antibodies did not provide reliable results, potentially due to issues with antibody specificity (not shown). Instead, staining for total ERK and analyzing nuclear:cytoplasmic (N:C) ratios proved to be a highly reproducible and sensitive way to assess ERK activity. Staining of fixed spheroids with ERK (Figures 1E, F) and analysis of N:C ratios revealed a significant increase in active, nuclear ERK in higher stiffness culture conditions in both MCF7 (Figure 1G) and HCC1954 (Figure 1H) spheroids. Moreover, the levels of active ERK were higher in cells within the outer region of the spheroid (in contact with the ECM) compared to those inside (Figures 1G, H; right graphs). To confirm these findings, we generated MCF7 cells stably expressing a GFP-tagged ERK-KTR activity reporter which undergoes nuclear to cytoplasmic transition upon activation of ERK (de la Cova et al., 2017). Analysis of fixed GFP-ERK-KTR expressing spheroids co-stained for Ki67 (a marker of proliferating cells) demonstrated a significant increase in activity of ERK in higher stiffness collagen scaffolds (Supplementary Figures S1C, E) that correlated with a reduction in proliferation with increasing stiffness (Supplementary Figure S2D). Treatment of spheroids with U0126 significantly reduced proliferation and ERK activity, confirming the contribution of ERK to proliferation in this model (Supplementary Figures S2C, D).

To determine the key upstream receptor mediating ERK activity in this context, we treated HCC1954 cells with a range of inhibitors targeting EGFR and integrins. Out of the inhibitors tested, AG1478 (EGFR inhibitor) and BTT3033 (α2β1 integrin inhibitor) both resulted in a significant reduction in ppERK as measured by Western blotting (Supplementary Figure S3A) and in spheroids in all stiffnesses of collagen (Supplementary Figures S2B, C). Interestingly, ERK N:C ratios were significantly reduced in both outer and inner cells of spheroid by both EGFR and α2β1 inhibition (Supplementary Figure S3D) suggesting that disruption of cell-ECM interactions in outer cells also cascades to reduce ERK activity in neighboring cells. These reductions in ERK activity also correlated with significant reductions in nuclear counts in spheroids in gels of all stiffnesses (Supplementary Figure S3E).

These findings demonstrate that cell proliferation is suppressed in high stiffness collagen gels in two different breast cancer cell line spheroid models, but that this negatively correlates with an enhancement in ERK activity.

### 3.2 ERK activity is required for invasion in lower stiffness ECM

**FIGURE 2:**
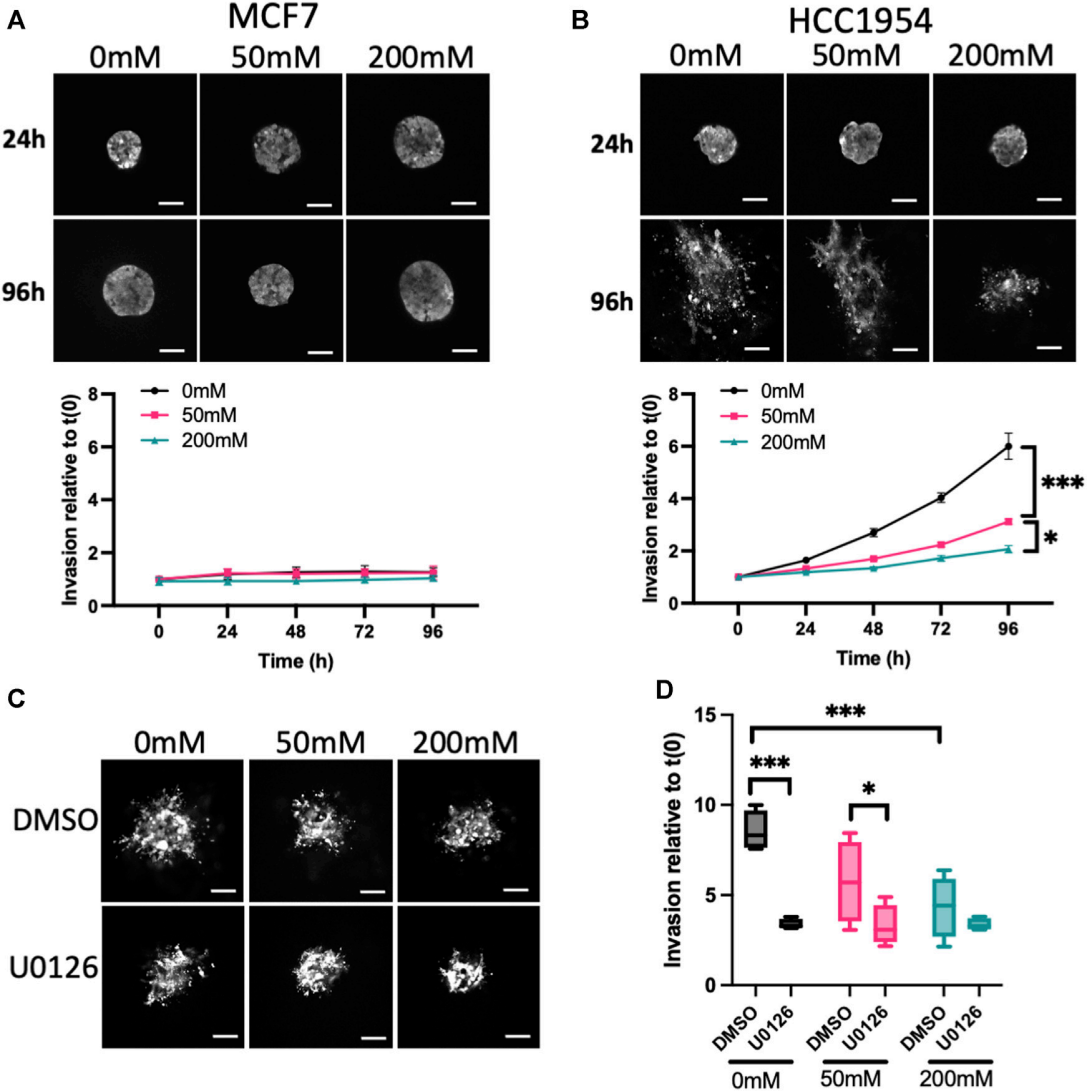
ERK activity anti-correlates with stiffness-dependent invasion. **(A, B)** Representative images of MCF7 **(A)** and HCC1954 **(B)** cell spheroids expressing LifeAct-GFP embedded in collagen gels stiffened using ribose (0, 50, and 200 mM). Live cells imaged every day over 96 h. Images from 24 to 96 h are shown. Graphs beneath images show quantification of invasion relative to time 0. **(C)** Representative images of live LifeAct-GFP expressing HCC1954 cell spheroids treated with DMSO or U0126 (1 μM) at 72 h post-embedding in collagen gels of differing stiffness. **(D)** Quantification of invasion at 72 h from all conditions. Data shown is from one experiment with 10 spheroids per condition, representative of 3 independent experiments. Mean and 25/75th percentile with min/max are shown in graphs. *p < 0.05, ***p < 0.001. Scale bars are 20 μm.

To further investigate other potential functional consequences of increased mechano-signaling in higher stiffness ECM, we monitored invasion of spheroids using LifeAct-GFP to define spheroid boundaries over time. MCF7 cells did not invade over the 5-day period of imaging (Figure 2A), in line with previous reports (Ziegler et al., 2014). However, HCC1954, an invasive cell line, exhibited significantly reduced invasion in higher stiffness collagen gels compared to unstiffened (Figure 2B). Invasion of HCC1954 spheroids in 0 and 50 mM ribose stiffened gels was ERK-dependent, as evidenced by reduced invasion in samples treated with U0126 (Figures 2C, D). However, ERK inhibition had no effect on the minimal invasion seen in 200 mM stiffer gels (Figures 2C, D). To understand whether α2β1 integrin and EGFR-dependent ERK activation also contributed to invasion, HCC1954 cell spheroids were treated with AG1478 and BTT3033 and invasion quantified after 72 h. Both inhibitors significantly reduced invasion in all stiffnesses, with AG1478 showing greater inhibition than BTT3033 (Supplementary Figure S3F).

This data collectively demonstrates that higher stiffness collagen suppresses spheroid proliferation and invasion, and that both phenotypes require active ERK, EGFR and α2β1 integrins.

### 3.3 Higher ECM stiffness increases YAP activity but suppresses membrane protrusions

**FIGURE 3:**
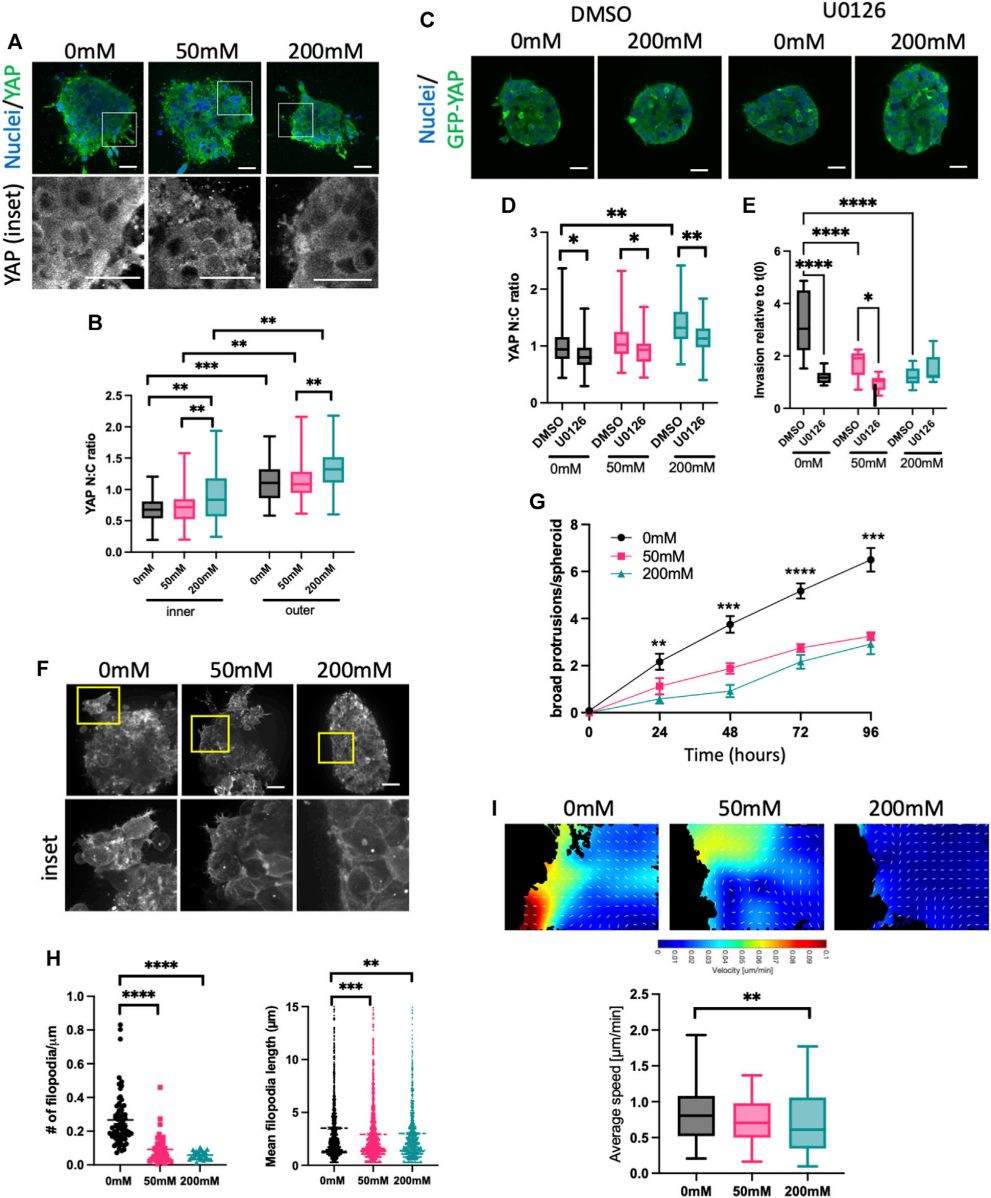
High stiffness ECM activates YAP but reduces F-actin protrusions. **(A)** Representative images of HCC1954 cell spheroids in collagen gels stiffened with ribose (0, 50, and 200 mM), fixed after 72 h and stained for nuclei (blue) and YAP (green). Boxed areas in top panels shown as higher magnification YAP inset panels below. **(B)** Quantification of YAP nuclear:cytoplasmic (N:C) ratio in inner and outer cells of spheroid from images as in **(A)**. **(C)** Representative images of MCF7 GFP-YAP (green) spheroids embedded in 0 mM or 200 mM ribose stained live with Hoechst (blue). Spheroids were treated with DMSO or U0126 (1 μM). **(D)** Quantification of YAP N:C ratio from images as in **(C)**. **(E)** Quantification of invasion of MCF7 GFP-YAP spheroids embedded in 0, 50 or 200 mM ribose. Spheroids were treated with DMSO or U0126 (1 μM) and invasion quantified live over 96 h data shows 96 h time point relative to time 0 h. **(F)** Representative images of LifeAct-GFP expressing HCC1954 cell spheroids in collagen gels stiffened with ribose (0 mM, 50 mM and 200 mM) imaged live. Yellow boxes denote inset panels showing protrusions. **(G)** Protrusions/spheroid over time from conditions and images as in **(F)**. **(H)** Filopodia number/μm spheroid boundary (left) and filopodia length (right) quantified from images as in **(E)**. **(I)** Representative images of particle imaging velocimetry (PIV) temporal maps of F-actin dynamics from movies of LifeAct-GFP HCC1954 cell spheroids (as in **(C)**; edge of spheroid shown). Graph (right) shows quantification of actin flow speed quantified from PIV analysis. Data shown is from one experiment with 10 spheroids per condition, representative of three independent experiments. Mean and 25/75th percentile with min/max are shown in graphs. *p < 0.05, **p < 0.01, ***p < 0.001, ****p < 0.0001. Scale bars are 20 μm.

Our surprising findings that ERK activity was increased by collagen stiffening, but this negatively correlated with proliferation and invasion, prompted us to assess another well documented mechano-sensitive signaling protein, YAP. As is the case for ERK, YAP is transported into the nucleus upon activation, and activity can be quantified by analyzing the N:C ratio. HCC1954 spheroids fixed and stained for YAP showed significantly higher nuclear YAP in 20 mM ribose stiffened gels compared to 0 and 50 mM (Figures 3A, B). As with ERK, higher YAP activity was seen in the outer cells in contact with the ECM vs. inner cells (Figure 3B). Further confirmation that nuclear, active YAP was dependent upon external forces was evidenced by a reduction in YAP N:C in spheroids subjected to laser ablation of surrounding ECM fibers using multiphoton microscopy (Supplementary Figure S4A).

To determine whether YAP was sufficient to enhance invasion, we generated MCF7 cells stably overexpressing GFP-YAP. Western blotting demonstrated very low endogenous YAP in MCF7, with GFP-YAP expressed at similar levels to that seen in HCC1954 cells (Supplementary Figure S4B). YAP N:C ratios in GFP-YAP MCF7 spheroids were higher in 200 mM ribose stiffened collagen gels compared to lower stiffnesses and were significantly higher in outer vs. inner cells of spheroids in all stiffness conditions (Supplementary Figure S4C), confirming findings seen with endogenous YAP in HCC1954 spheroids (Figure 3B). GFP-YAP overexpressing MCF7 cells also exhibited significantly higher nuclei numbers after 96 h of growth in 0 and 50 mM ribose gels, but not 200 mM (SSupplementary Figures S4D, E). Overexpression of GFP-YAP did not alter localization of E-cadherin at cell-cell adhesions compared to GFP control spheroids (Supplementary Figure S4F). However, enhanced invasion was seen in GFP-YAP expressing spheroids compared to GFP alone controls MCF7 in 0 and 50 mM ribose gels and this was coupled with an increase in broad multicellular protrusions (Supplementary Figures S4G, H). Moreover, siRNA mediated depletion of YAP from HCC1954 cells resulted in reduced broad multicellular protrusions and invasion in 0 and 50 mM ribose stiffened gels (Supplementary Figures S4I–K). These findings collectively demonstrate that YAP is sufficient and required to promote spheroid proliferation and invasion in lower stiffness 3D collagen gels.

Several studies have suggested that ERK operates upstream of YAP in the biomechanical signaling cascade (Yu et al., 2015; Qin et al., 2019; Shi et al., 2020). We tested whether this was the case in our model by treating MCF7 GFP-YAP spheroids with DMSO or U0126. U0126 treatment significantly reduced YAP activity (N:C ratio) in spheroids embedded in all stiffnesses (Figures 3C, D). Moreover, ERK inhibition led to a significant reduction in YAP-dependent invasion in MCF7 GFP-YAP spheroids in 0 and 50 mM ribose stiffened collagen gels (Figure 3E), mirroring that seen in HCC1954 cell spheroids (Figures 2C, D). This data indicates that YAP activity requires activity of ERK, and that YAP-dependent invasion in lower stiffness 3D collagen is also ERK-dependent.

YAP activity has been proposed to be both a regulator of (Lamar et al., 2012; Qiao et al., 2017) and regulated by (Aragona et al., 2013; Driscoll et al., 2015) the organization and dynamics of the F-actin cytoskeleton. To determine whether YAP activity correlated with changes to F-actin organization, we performed high resolution live imaging of LifeAct-GFP expressing HCC1954 spheroids in 0, 50, and 200 mM ribose stiffened collagen gels (Figure 3F; Supplementary Videos S1–S3). Distinct forms of peripheral F-actin containing protrusions were evident, particularly in spheroids in lower stiffness collagen gels. Numbers of large protrusions from the spheroid boundary formed by three or more cells increased over time and were significantly higher in 0 mM compared to 50 or 200 mM ribose collagen gels (Figures 3F, G). Finer, highly dynamic filopodia like protrusions were also seen in most outer cells in spheroids in 0 mM ribose, but the number and length of these filopodia decreased with increasing ECM stiffness (Figures 3F, H). Further analysis of the dynamics of actin flow within outer cells using particle imaging velocimetry [PIV (Yolland et al., 2019)] revealed a significant reduction in actin flow speed in cells within 200 mM vs. 0 mM ribose gels (Figure 3I).

### 3.4 YAP, ROCK and MMP activity are required for invasion and ECM remodeling in lower stiffness ECM

**FIGURE 4:**
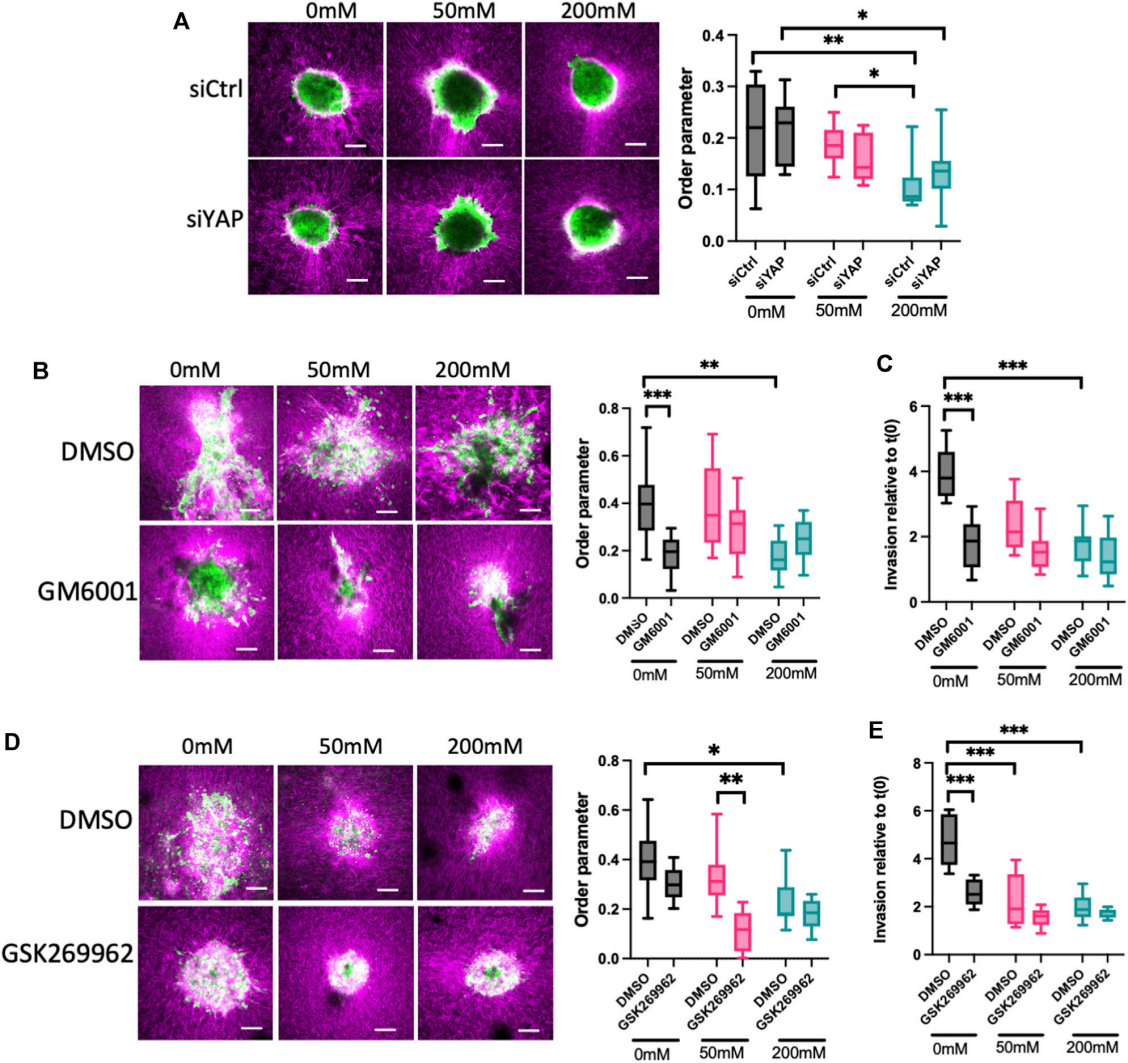
ROCK and MMPs contribute to ECM remodeling and invasion in low stiffness 3D collagen scaffolds. **(A)** Representative images of LifeAct-GFP HCC1954 cells spheroids (green) embedded in cy3-labelled collagen gels (magenta). Cells were treated with control or YAP targeted siRNA prior to embedding. Graph on right shows analysis of order parameter (alignment) of collagen fibres in all conditions from images shown measured 24 h post-embedding. **(B)** Representative images of LifeAct-GFP HCC1954 cell spheroids (green) embedded in cy3-labelled collagen gels (magenta) treated with DMSO or GM6001 (pan-MMP inhibitor; 10 μm) imaged 72 h post-embedding. Graph on right shows analysis of order parameter (alignment) of collagen fibres in all conditions from images shown. **(C)** Quantification of invasion from conditions shown in **(B)** at 72 h post-embedding relative to time 0 h. **(D)** Representative images of LifeAct-GFP HCC1954 cell spheroids embedded in cy3-labelled collagen gels (magenta) treated with DMSO or GSK269962 (ROCK inhibitor; 2 μm) imaged 72 h post-embedding. Graph shows quantification of order parameter (alignment) of collagen fibers in all conditions from images shown in **(D)** measured 72 h post-embedding **(E)** Quantification of invasion from conditions shown in **(D)** at 96 h post-embedding relative to time 0 h. Data shown is from one experiment with 10 spheroids per condition, representative of three independent experiments. Mean and 25/75th percentile with min/max are shown in graphs. *p < 0.05, **p < 0.01, ***p < 0.001, ****p < 0.0001. Scale bars are 20 μm.

Our data thus far show that ERK is required to fully activate YAP, and that YAP contributes to F-actin dynamics that correlate with invasion in lower stiffness collagen gels. To better understand the relationship between spheroid phenotypes and ECM organization, we sparsely labelled collagen with Cy3 dye prior to polymerization and visualized collagen and HCC1954 cell spheroids simultaneously. Analysis of collagen fiber organization using AFT revealed a significant reduction in alignment of collagen fibers induced by spheroids in 200 mM ribose stiffened gels compared to 0 and 50 mM (Figure 4A). However, silencing of YAP in these cells did not alter organization of collagen fibers in any stiffness (Figure 4A), indicating YAP is not sufficient to remodel the collagen fibers to permit spheroid growth and invasion.

Matrix metalloproteinases (MMPs) are a key class of enzymes that target and degrade specific ECM proteins and have been widely shown to be important in invasion of epithelial-like breast cancer cells (Winkler et al., 2020). We therefore tested whether MMPs were required for the ECM alignment and subsequent invasion in our model. Treatment of HCC1954 cell spheroids with the pan-MMP inhibitor GM6001 resulted in a significant reduction in the alignment of collagen fibers in 0 mM ribose gels, but not 50 or 200 mM ribose stiffened matrices (Figure 4B). Moreover, this suppression of ECM remodeling led to a functional reduction in invasion in 0 mM gels but had no effect on the reduced invasion seen in 50 and 200 mM ribose stiffened gels (Figures 4B, C). This data indicated that MMP activity is required to reorganize the collagen fibers to permit invasion under lower stiffness conditions. To further determine whether this depended on F-actin based contractility to drive the observed protrusions, cells were treated with the ROCK inhibitor GSK269962 to suppress actomyosin-based contractility. Inhibition of ROCK led to a significant reduction in collagen alignment in 50 mM ribose stiffened gels compared to DMSO treated controls, but no changes were observed under other stiffness conditions (Figure 4D). Interestingly, ROCK inhibition suppressed invasion in low stiffness collagen gels to the same degree as MMP inhibition but had no effect on invasion of spheroids into 50 and 200 mM ribose stiffened collagen gels (Figures 4D, E).

These findings collectively demonstrate that invasion of breast cancer spheroids under low stiffness ECM conditions requires YAP activity, and this in turn depends on actomyosin contractility and ECM remodeling by MMPs to create a matrix that is permissive for migration.

## 4 Discussion

A relationship between ECM stiffness and breast cancer progression has been well documented, but the diversity of models, both 2D and 3D, used to investigate this phenomenon have led to often conflicting conclusions regarding roles for matrix biomechanics in mechanosensitive signaling and subsequent functional effects. Here we have used two different human breast cancer cell lines—both “epithelial-like” in nature—to investigate how increasing stiffness of collagen scaffolds alters ERK and YAP activation in cells within 3D spheroids and their contributions to functional phenotypes. Using a variety of orthogonal approaches, our data provide evidence that ERK and YAP are both activated in response to increasing stiffness in inner and outer cells of spheroids. However, this is not sufficient in tumor cells alone to drive proliferation or invasion when ECM stiffness is high. We propose that activity of these key mechano-sensitive pathways may instead play alterative roles in “priming” cells either to promote survival (for example, following chemo-toxic stress) and/or drive a delayed transcriptional program to remodel the ECM for permissive invasion.

Our study focuses on spheroids in native 3D collagen gels, stiffened with ribose. We chose to use collagen I as our model as is the most abundant protein within the stromal compartment surrounding solid tumors. This contrasts with Matrigel, which has been widely used by others, but as a basement membrane composite does not recapitulate the topography or predominant ECM composition of tumor stroma which can impact on interpretation (Berens et al., 2015). Ribose has also been shown to increase stiffness and viscosity without altering organization and ligand binding sites for cells (Mason et al., 2013; Mason and Reinhart-King, 2013; Elosegui-Artola et al., 2023), allowing the investigation of stiffness alone in controlling tumor cell behavior. Much of the previous work studying ERK and YAP activity in response to stiffness—and functional consequences therein—have been conducted in cells on 2D surfaces, often at non-physiological stiffnesses (>100 kPa), and which do not recapitulate the 3D environment in solid tumors. Our data agrees with other studies demonstrating that increased 3D collagen stiffness can suppress proliferation and invasion in other cancer types (Berger et al., 2017; Staneva et al., 2018; Berger et al., 2020; Mahajan et al., 2021).

To our knowledge, ours is the first study to demonstrate that both ERK and YAP are activated in human breast cancer cell spheroids in response to ECM stiffness, and that ERK lies upstream of YAP activity in this context. This would agree with a recent study in normal breast epithelial cells showing YAP does not control ERK activity (Farahani et al., 2021). A recent study demonstrated that breast cancer organoids show reduced collective invasion and increased active YAP in stiffer collagen gels (Koorman et al., 2022). The same study suggested that collectively invading cells increase local stiffening of collagen through secretion of the collagen crosslinker lysyl oxidase-like 3 and speculate that this may enhance local YAP activity and actomyosin contractility to stimulate local invasion (Koorman et al., 2022). Our study confirms that both actomyosin contractility and MMPs are required for collective invasion of spheroids under lower stiffness ECM conditions. Highly crosslinked collagen very likely results in ECM that is more refectory to degradation by MMPs, as has been suggested in a recent study (Vaez et al., 2023), and this may partially explain the phenotypes we report here under high stiffness conditions. Interestingly, our data indicates that ROCK inhibition only significantly reduces collagen alignment under 50 mM ribose conditions, but that this is not sufficient to reduce invasion in the same cultures. Conversely, ROCK promotes invasion in 0 mM ribose gels but does not promote collagen alignment. Very few studies have assessed roles for MMPs and actomyosin in epithelial-like cancer spheroid invasion and matrix alignment. However, combined modelling and experimental data have shown non-linear crosstalk between these pathways indicating a more complex feedback mechanism exists in response to matrix stiffness (Ahmadzadeh et al., 2017). Importantly our data indicate that under the highest stiffness conditions measured in our study, neither actomyosin or MMPs are able to A recent pre-print reports on sorting of proliferative cells to the outside of spheroids in low stiffness hydrogels to mediate ECM remodeling and invasion (Cai et al., 2023), which may be reduced under high stiffness conditions. Analyzing the temporal relationship between ERK, YAP and actomyosin activation in live 3D cultures and the potential role for local secreted matrix modifiers would be important to explore in future to investigate this co-operativity further.

Our data provide strong evidence that YAP expression and activity is required to induce proliferation and invasion. However, our findings that higher levels of nuclear YAP in stiffer matrices correlate with reduced proliferation, ECM remodeling and invasion appear counterintuitive. One explanation, as previously suggested by others, is that YAP requires context-specific parallel signals to induce pro-tumorigenic phenotypes (Zhang et al., 2018). Thus, YAP may only be capable of inducing invasive behavior within a physically permissive matrix and higher ECM crosslinking may control alternative pathways that do not synergize with increased YAP activity. The changes to the phospho-proteome induced by stiffness in breast cancer models have yet to be explored but is an important future goal to understand potential co-operative signals with YAP absent under high stiffness conditions. Previous studies in cells on 2D surfaces have also suggested that sustained YAP activity can induce apoptosis (Chiba et al., 2016; LeBlanc et al., 2021). A higher rate of cell death in higher stiffness conditions was not visibly evident in our live cultures, but we cannot rule this out as a potential consequence of increased YAP activity in these spheroids.

Our study also demonstrates that EGFR and the collagen-binding integrin α2β1 are both required for activation of ERK irrespective of ECM stiffness, and likely also contribute to YAP activity. Both receptor types have previously been suggested to contribute to ERK activity in cancer cells in 2D environments of differing stiffness (Provenzano et al., 2009; Wang et al., 2021). Interestingly, our data shows that inhibiting both receptors reduced activity of ERK in both the outer and inner cells of spheroids. Given α2β1 integrins are collagen receptors, it may be surprising that they contribute to ERK activity in inner spheroid cells, where no ECM contact is present. One explanation for this might lie in our recent discovery that active α2β1 integrins are present at epithelial cell-cell adhesions where they contribute to junction stability (Howden et al., 2021), and previous studies showing colocalization between integrins and EGFR at these sites (Yu et al., 2000). Whilst this has yet to be assessed in cancer cell spheroids or tumors, it is possible that integrins also have functional roles in non-ECM binding cells in our current model. An alternative, or complementary, explanation may lie in signal propagation between cells. Previous studies have shown that ERK activity can propagate as pulsatile waves within sheets of non-transformed cells (Hiratsuka et al., 2020; De Simone et al., 2021; Gagliardi et al., 2021; Lin et al., 2022), although whether this also occurs in cancer spheroids or organoids remains unclear. Indeed, oncogenic mutations in the MAPK pathway can disrupt normal ERK dynamics, leading to sustained activation and cell cycle arrest (Aikin et al., 2020) and this may bypass any effect of mechanical stiffness on ERK activity (Farahani et al., 2021). Whilst the roles of ECM mechanics in dynamics of ERK remain to be explored, it is possible that the higher ERK activity in higher stiffness collagen we report here is due to sustained activation and thereby suppression of proliferation under these conditions. It would be important to multiplex ERK or YAP reporter probes with cell cycle reporters [such as FUCCI (Sakaue-Sawano and Miyawaki, 2014)] to gain a better understanding of the spatiotemporal relationships between stiffness, sustained signaling and proliferation. Notably, both integrins and cell-cell adhesion receptors can modulate upstream components in the Hippo pathway, such as NF2 and LATS1/2, leading to YAP activation in cancer cells (Dey et al., 2020). Whilst roles for these pathways in controlling ERK activation remain to be defined, additional analysis of these upstream regulators in conjunction with ERK and YAP activity may help to define integrin and cell-cell adhesion receptors contributions to spatial activation of these key pathways.

Our data also reveals suppression of F-actin dynamics in cancer cell spheroids in higher stiffness 3D collagen. Several studies have documented differences in F-actin architecture in cells on 2D vs in 3D environments (Caswell and Zech, 2018; Tran and Kumar, 2021), and whilst stiffer 2D surfaces can induce F-actin stress fiber assembly, these are not evident in 3D cancer cell spheroids. Our data agrees with a previous study suggesting higher stiffness 3D matrices reduce F-actin protrusions and increase spheroid compaction (Taubenberger et al., 2019). However, to our knowledge, ours is the first study to image F-actin dynamics in live 3D cancer cell spheroids in collagen matrices. The presence of collectively invading multicellular protrusions, with multiple filopodia, indicates that the latter may provide a sensing mechanism for cells to explore the surrounding 3D fibrillar topography, as we and others have previously suggested (Jacquemet et al., 2017; Pfisterer et al., 2020). It remains unclear whether these filopodia contain integrins to enable cell adhesion and further traction force generation. Adhesions are difficult to image in 3D matrices as they are generally smaller than in cells on 2D, and high-resolution 3D imaging remains challenging. However, our data demonstrates that ROCK is required for these protrusions, indicating they are actomyosin-dependent structures and likely therefore contribute to ECM alignment and invasion. Protrusions may also act to locally secrete MMPs, or localize membrane bound proteases such as MT1-MMP to drive invasion (Ferrari et al., 2019). Higher stiffness collagen appears to elicit compression forces on outer cells, resulting in suppression of filopodia and multicellular protrusions, which likely leads to suppression of invasion. It is possible these compressive forces override the higher ERK and YAP activity seen in these spheroids, preventing pro-tumorigenic effects. Future studies to understand the spatiotemporal adhesion and F-actin related pathway changes in spheroids or organoids in higher stiffness conditions may help to reveal the nature of these dominant mechanisms to explain the phenotypes we report here.

### 4.1 Limitations

Our study focused on the use of cancer cell spheroids alone within mechanically tuned collagen scaffolds as a simplified model to test our hypotheses. We chose to pre-form spheroids using the hanging drop method, which has been widely used by others. However, pre-assembling spheroids in ECM-free conditions may lead to different findings as compared to seeding single cells in collagen gels and allowing them to form spheroid-like clusters over time. The latter may mimic the *de novo* formation of a solid tumor, but itself has limitations in terms of timing of culture formation (likely days to weeks) and the impact this would have on the integrity of the collagen scaffolds. Moreover, spheroids embedded in collagen wells do not recapitulate the full complexity of the disease. Several studies have focused on the co-culture of cancer cells with cancer-associated fibroblasts (CAFs), and the contribution of CAFs to a more fibrotic like-tumor microenvironment has been well documented (Jang and Beningo, 2019). CAFs produce a broad range of ECM proteins including fibronectin, laminins and tenascins that contribute to modulating phenotypic changes, (Popova and Jucker, 2022). Our use of 3D collagen I hydrogels here may initiate some ECM synthesis by the cancer cells, but the lack of other ECM proteins is likely to impact on integrin-dependent signals that may further modulate the proliferative and invasive behavior of spheroids. Patient-derived organoids can better reflect the complex heterogeneity of breast cancers (Gunti et al., 2021) but still lack the vascular flow and immune compartments, which have proven very challenging to mimic *in vitro* (Yuan et al., 2022). Xenograft and transgenic animal models are also very widely used in cancer biology, and whilst they provide an *in vivo* like environment, they often also fail to recapitulate human disease, and are not readily amendable to high resolution imaging or mechanistic studies (Ireson et al., 2019). Future efforts to build better *in vitro* preclinical models of human disease are critical to understand cancer progression and aid drug discovery.

We also chose here to stiffen collagen gels using ribose, rather than also increasing stiffness using higher collagen concentrations. Both approaches stiffen ECM to similar degrees, but the latter also increases integrin ligand binding sites and reduces pore size, which can influence interpretation. Given our findings regarding the importance of integrins in mediating mechanically induced signaling changes in spheroids, extending our study to higher density collagen matrices might represent an interesting comparison in future. A potential consideration of using ribose is the secondary effects that introduction of this sugar may have on cell metabolism and resulting phenotypes. Whilst a recent study showed no effect of ribose crosslinking on these processes in other cell types (Gouldin et al., 2022) we cannot rule out effects on the cells used in the current study. A further limitation in interpretation of our data is that absolute measurements of collagen gel stiffness over time was not performed, limiting our understanding of the mechanical changes occurring due to remodeling by the spheroids. Measurement of mechanical properties of 3D hydrogels ranges from the use of AFM, rheology, and compression analysis (Gandin et al., 2021), but gaining spatiotemporal quantitative measurements of changing stiffness remains challenging given the tools available. A recent demonstration of the use of Brillouin microscopy coupled with lightsheet imaging provides one potential means to quantify relative mechanical changes to both spheroid and ECM simultaneous with analysis of signaling or phenotypic changes (Bevilacqua et al., 2023). It would be interesting to explore the use of this technology in future models to understand the interplay between mechanical changes and tumorigenesis in greater detail.

## Data Availability

The raw data supporting the conclusions of this article will be made available by the authors, without undue reservation.
