# Supplementary figures and images for: Extracellular matrix stiffness activates mechanosensitive signals but limits breast cancer cell spheroid proliferation and invasion
Source: Front Cell Dev Biol. 2023 Dec 6;11:1292775. doi: 10.3389/fcell.2023.1292775 (PMC10731024; doi:10.3389/fcell.2023.1292775)

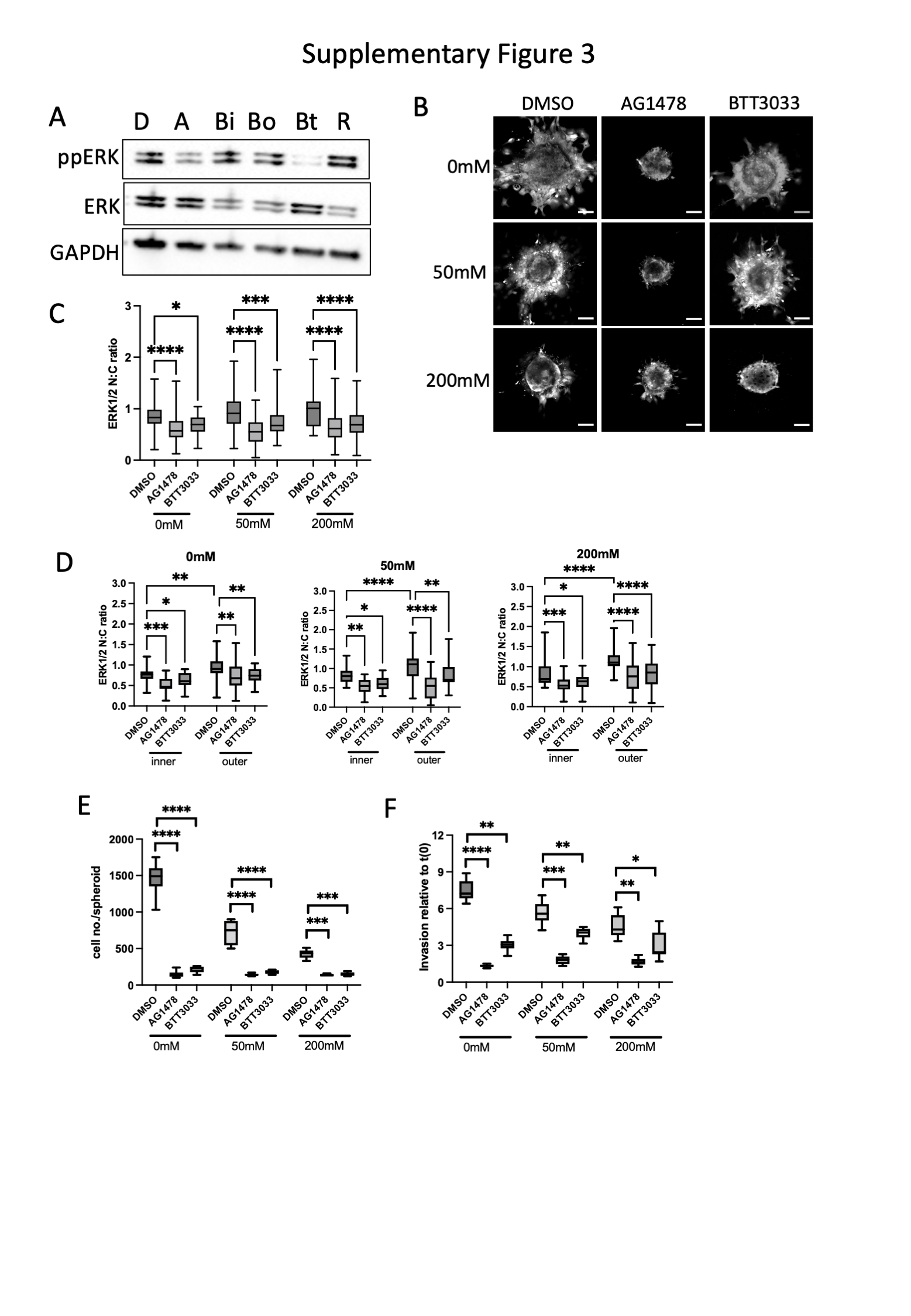

Supplement: Supplementary file 1 [file Image3.TIFF]

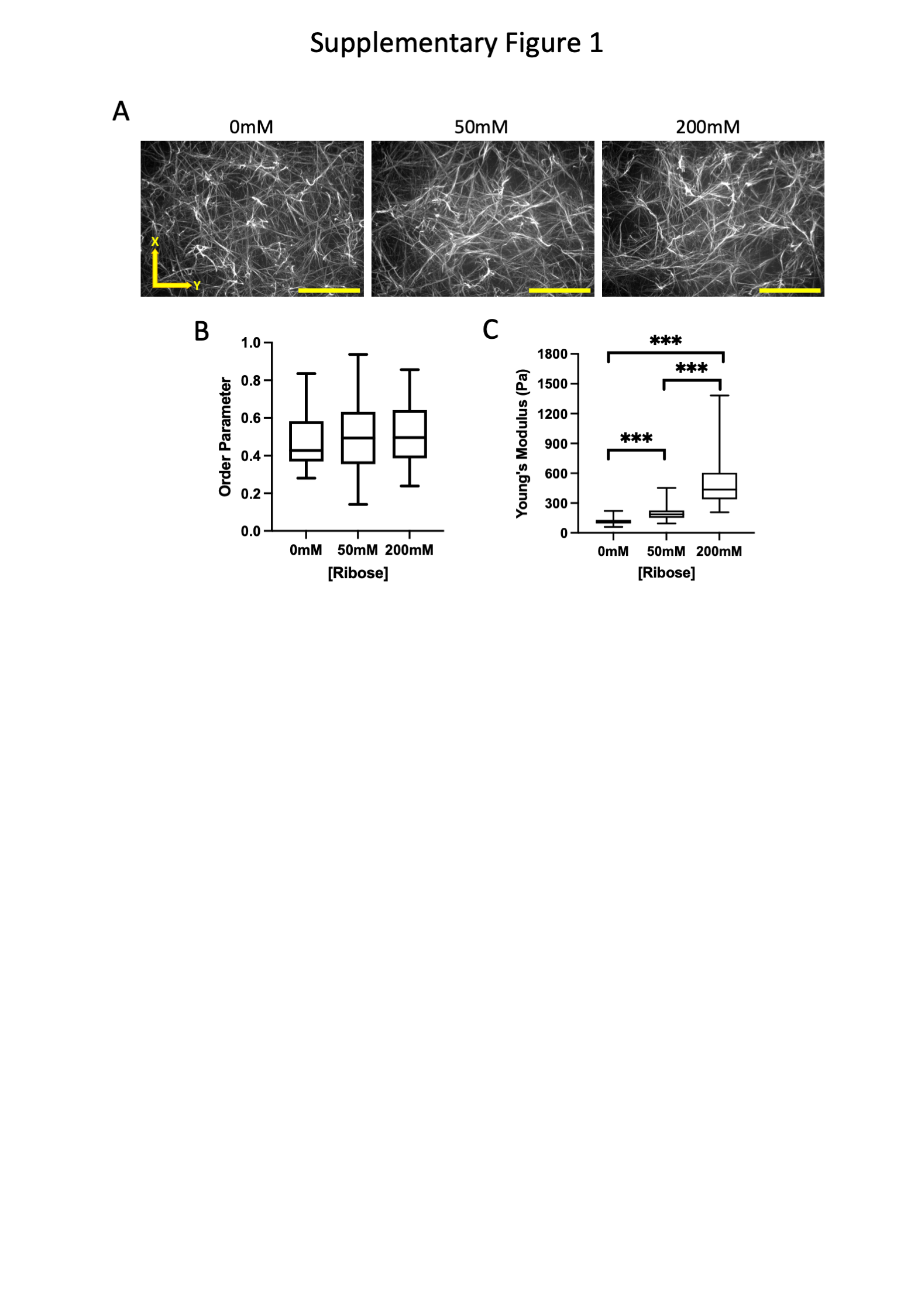

Supplement: Supplementary file 3 [file Image1.TIFF]

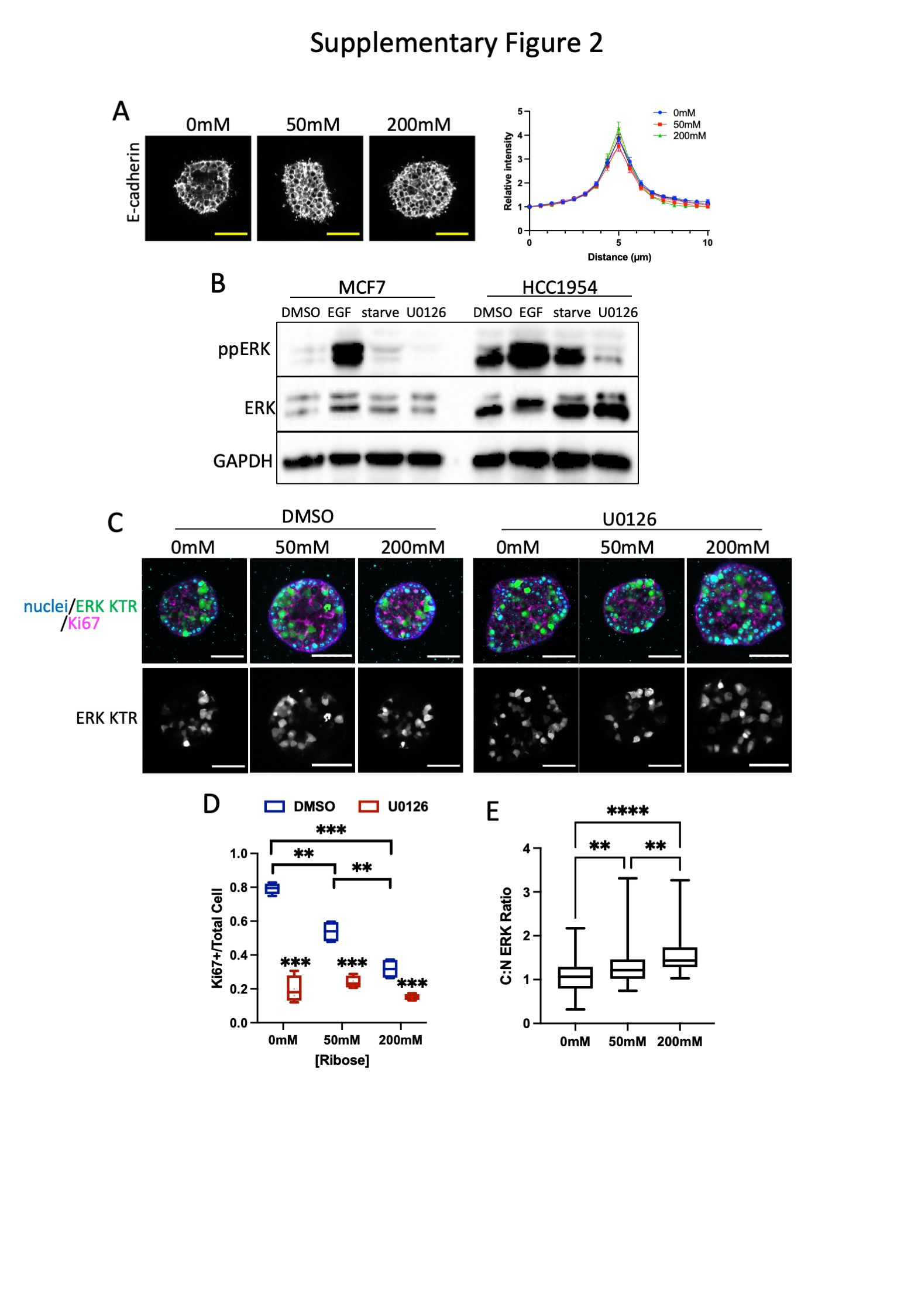

Supplement: Supplementary file 6 [file Image2.TIFF]

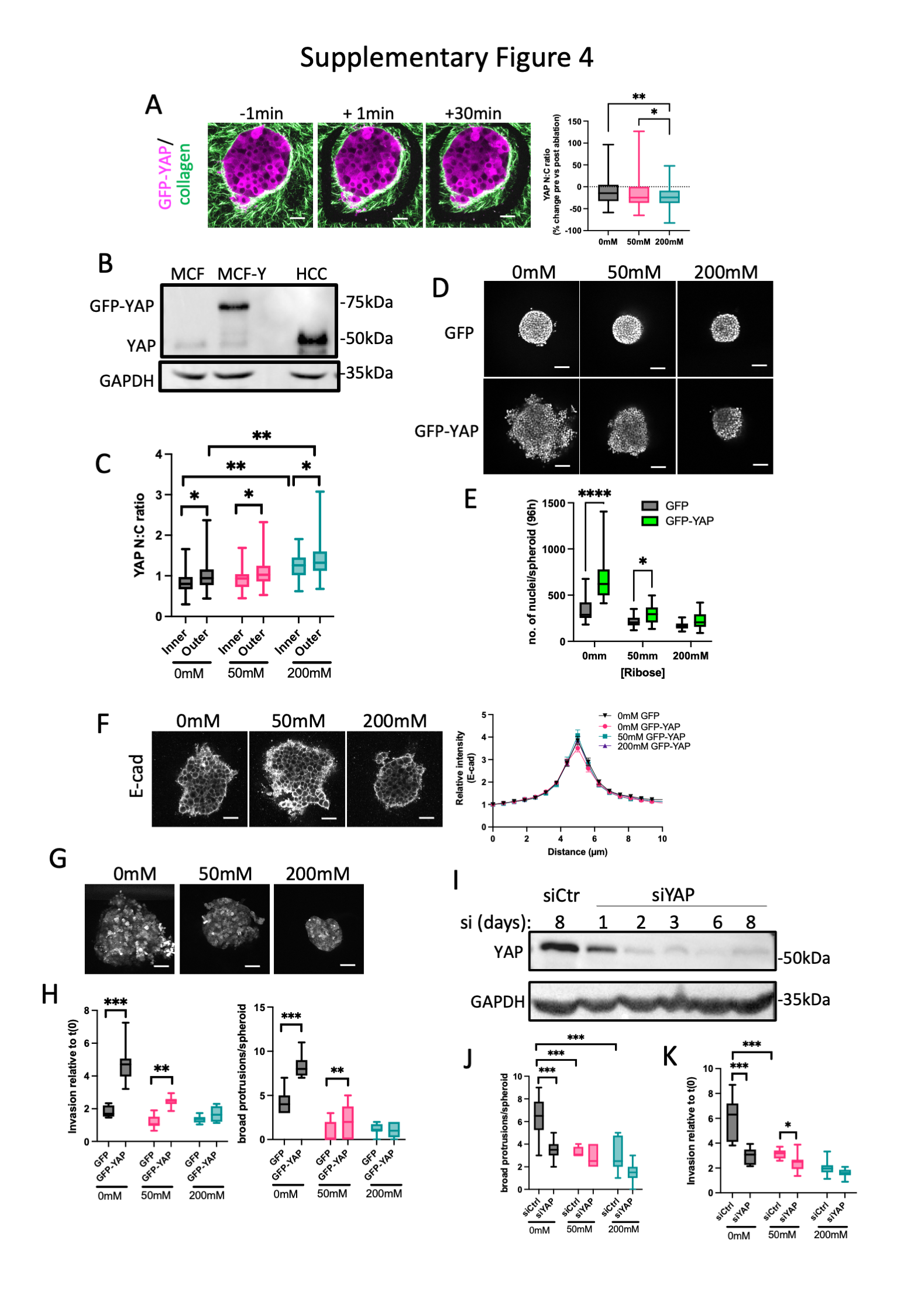

Supplement: Supplementary file 7 [file Image4.TIFF]
